# Orthopedic Surgeons’ Views of Hyaluronic Acid Formulations in the Management of Knee Osteoarthritis: A Questionnaire-Based Cross-Sectional Study

**DOI:** 10.3390/medicina57090990

**Published:** 2021-09-19

**Authors:** Shahd Alnasser, Fatima AlHussain, Hassan Asiri, Abdullah Almutairi, Hisham Alsanawi, Anas AR Altamimi, Yousif A. Asiri, Omar A. Almohammed, Yazed AlRuthia

**Affiliations:** 1 Department of Clinical Pharmacy, College of Pharmacy, King Saud University, P.O. Box 2454, Riyadh 11451, Saudi Arabia; 438203348@student.ksu.edu.sa (S.A.); 437104272@student.ksu.edu.sa (H.A.); 437101323@student.ksu.edu.sa (A.A.); yasiri@KSU.EDU.SA (Y.A.A.); oalmohammed@ksu.edu.sa (O.A.A.); 2 Department of Pharmacoeconomics and Drug Pricing, Saudi Food and Drug, Authority, P.O. Box 84983, Riyadh 11681, Saudi Arabia; fhhussain@sfda.gov.sa; 3 Department of Orthopedic Surgery, College of Medicine, King Saud University, P.O. Box 3145, Riyadh 12372, Saudi Arabia; halsanawi@KSU.EDU.SA; 4 Department of General and Special Surgery, Faculty of Medicine, Hashemite University, P.O. Box 921113, Amman 11192, Jordan; tamimi8080@yahoo.com; 5 Pharmacoeconomics Research Unit, Department of Clinical Pharmacy, College of Pharmacy, King Saud University, P.O. Box 2454, Riyadh 11451, Saudi Arabia

**Keywords:** hyaluronic acid, orthopedics, osteoarthritis, pain management, Middle East

## Abstract

*Background and Objectives***:** Multiple hyaluronic acid (HA) products were approved and marketed to manage osteoarthritis (OA). Although these products are widely prescribed by orthopedic surgeons to manage OA, especially knee OA, the therapeutic value of these products is highly uncertain. Few studies with significant limitations in their designs have indicated positive outcomes among OA patients treated with HA; however, their results were inconclusive. Thus, we aimed to explore the therapeutic value of different HA products in alleviating knee OA pain and improving patients’ physical function from the orthopedic surgeons’ perspective. *Materials and Methods*: This was a questionnaire-based cross-sectional study in which practicing orthopedic surgeons in two countries (e.g., Saudi Arabia and Jordan) were invited to participate. The 10-item, newly developed questionnaire inquired about the respondents’ sociodemographic characteristics (e.g., age, gender, country, years of experience), and their opinions regarding the efficacy of HA products in the management of OA (e.g., efficacy in improving mobility and alleviating pain). *Results*: Out of the 200 orthopedic surgeons who were invited to participate, 122 (61%) filled out the questionnaire. Most of the respondents were from Saudi Arabia (58%), aged 35 to 55 years (68%), had at least 10 years of experience (69%), and male (98%). About 80% of the respondents reported prescribing HA, such as Hyalgan^®^, Orthovisc^®^, Hyalubrix^®^, and Crespine Gel^®^. About 66% of the respondents believed that HA was moderately to highly effective in managing knee OA, and 34% believed that HA was either ineffective or mildly effective. Pain at the site of injection (44.3%) and rash or local skin reactions (22.1%) were the most commonly reported adverse events. *Conclusions*: The variations in the formulation of different HA brands (e.g., molecular weight and cross-linking) did not seem to offer any therapeutic advantage. HA might have value in the management of knee OA; however, its value is highly uncertain and necessitates more well-designed studies to further examine its therapeutic value.

## 1. Introduction

Hyaluronic acid (HA) is an unbranched, anionic, linear chain of polysaccharides with a high molecular weight (~1000 kDa). It is commercially available in different pharmaceutical dosage forms for multiple indications, such as dry eye, osteoarthritis, prevention of post-surgery tissue adhesion, and facial augmentation [1]. The HA that is naturally produced by the body hydrates and lubricates the joints and tissues [1,2]. It has a short half-life and a high turnover rate. Therefore, an equilibrium state between synthesis and degradation is maintained [2]. However, aging results in a disruption of the HA homeostasis [3]. HA deficiency is associated with a myriad of symptoms that are based on the affected organs. For example, HA deficiency in the skin and eyes results in itchiness and dry eye/skin [4]. Additionally, exogenous HA is administered to alleviate pain due to tissue degeneration in large joints, such as the knees and hips [4,5].

Knee osteoarthritis (OA) is prevalent among adults aged ≥60 years with a global prevalence and incidence rates of 16% and 203 per 10,000 person-years, respectively [6]. In Saudi Arabia, the prevalence of knee OA is believed to be high, ranging from 24 to 60.9%, with higher incidence rates among older adults (i.e., ≥60 years) and obese people [7,8]. HA preparations for the management of osteoarthritis (OA) were proven to be safe and effective in reducing pain and enhancing physical function, albeit the magnitude of their clinical benefit is variable [9]. Since naturally occurring HA has a very short half-life, exogenous HA formulations were produced using different biotechnological techniques to increase their half-life and hopefully their efficacy [10]. Additionally, it is believed that the cross-linking of HA improves its mechanical, rheological, and swelling properties, as well as reduces its degradation rate [11]. The more similar the HA preparations are to the naturally occurring HA in synovial fluid in terms of their viscosity and other molecular properties, the more likely these exogenous HA preparations are thought to work in a similar way to the naturally occurring HA. Therefore, different HA products’ manufacturers try to imitate the molecular and rheological properties of the naturally occurring HA in synovial fluid [12].

Although intra-articular HA injections are thought to have some value in comparison to a placebo [9], some studies have found no difference between HA and a placebo in the management of OA [13,14]. However, some studies showed that multiple HA injections can result in significant pain reduction and improvement in physical function in comparison to a single HA injection [14]. Moreover, combining platelet-rich plasma (PRP) with HA resulted in better outcomes in comparison to HA alone, which did not result in a significant reduction in OA pain or physical limitation [15]. In addition, the use of HA injections showed similar efficacy to corticosteroids in alleviating OA pain and improving physical function after three and six months of treatment, but the rate of topical side effects, such as joint swelling and stiffness, were higher with HA [16]. This was confirmed in another post-marketing surveillance study in Jordan that followed 84 patients with knee OA for nine months to evaluate the safety and efficacy of the intra-articular Crespine^®^ Gel injection (e.g., a hyaluronic acid product) by interviewing them at different points of time using the Western Ontario and McMaster Universities Arthritis Index questionnaires (WOMAC). Significant reductions in pain and stiffness scores were noted after five months of treatment, and only minor and transitional adverse events, such as swelling and redness of the knee, were reported [17]. The European Society for Clinical and Economic Aspects of Osteoporosis and Osteoarthritis (ESCEO) also recommends the use of intra-articular HA for OA treatment based on an extensive literature review of real-world evidence and surveys [9]. ESCEO reported in their recommendation that the use of HA could result in a 50% reduction in the use of analgesics, improvement in pain and physical function, and a delay in the need for a total knee replacement surgery [9]. Nonetheless, another systematic review of 14 randomized controlled trials to assess the effectiveness of intra-articular HA in the management of knee OA found that HA only results in a modest pain reduction after the first six to eight weeks of therapy, and this effect fades and becomes doubtful at six months [18]. Additionally, the cost-effectiveness of different intra-articular HA products in comparison to disease-modifying osteoarthritis drugs, such as non-steroidal anti-inflammatory drugs (NSAIDs) and corticosteroids, is not well-established and can exceed the acceptable cost-effectiveness thresholds [19]. Therefore, the aim of this study was to explore orthopedic surgeons’ views on the efficacy and safety of intra-articular HA products in the management of knee OA in Saudi Arabia and Jordan.

## 2. Methods

### 2.1. Ethical Approval of the Study

The research proposal was reviewed and approved by the institutional review board of the College of Medicine at King Saudi University, Riyadh, Saudi Arabia (approval of research project no. E-20-4736).

### 2.2. Study Design and Population

This was a descriptive questionnaire-based cross-sectional study in which orthopedic surgeons with at least one year of experience in orthopedic surgery who frequently see OA patients in Saudi Arabia and Jordan were invited to participate using a convenience sampling technique. A list of 200 orthopedic surgeons with their emails and contact information who met the inclusion criteria was created by the fifth and sixth authors, who are practicing orthopedic surgeons in Saudi Arabia and Jordan, respectively. An invitation to participate with the link to the online questionnaire was sent to the potential participants via WhatsApp^®^ and email.

### 2.3. Questionnaire Development

In order to explore orthopedic surgeons’ views regarding the efficacy and safety of intra-articular HA products in the management of knee OA pain, a 10-item questionnaire was developed (Appendix A). The first six items inquired about the sociodemographic characteristics of the participants (i.e., age, gender, the country where the participant was practicing, where the postgraduate training was received, years of experience, and whether the participant was primarily practicing in a public or private healthcare setting). The other four items inquired about whether the participant was prescribing HA injections for the management of OA pain, the effectiveness of HA injections in the management of OA using a 5-point Likert scale (e.g., (1) not effective, (2) mildly effective, (3) moderately effective, (4) effective, and (5) highly effective), the HA products the participant was prescribing for knee OA, whether using these products resulted in any improvement in patient mobility and/or pain, and the observed side effects of HA products. The questionnaire’s content validity and face validity were checked by the authors. Pilot testing among 25 individuals was conducted to ensure the clarity and comprehensibility of the questionnaire. Only minor changes were made and the questionnaire had acceptable internal consistency (e.g., Cronbach’s alpha of 0.61) [20].

### 2.4. Statistical Analysis

Descriptive statistics using frequencies and percentages were conducted to present the findings of the study. All statistical analyses were performed using SAS^®^ version 9.4.

## 3. Results

Out of 200 orthopedic surgeons who were invited to participate, 122 (61%) consented to participate and filled out the questionnaire. About 60% of the respondents were from Saudi Arabia and 40% were from Jordan. The majority of the respondents were between 35 and 55 years of age (68%) and had at least 10 years of experience (68.85%). Only two respondents (1.64%) were females and the rest were males. Most of the respondents (68%) received their postgraduate training in the Middle East (e.g., Saudi Arabia, Jordan, Bahrain, Egypt, and Syria) and were practicing in public healthcare facilities (65.57%) (Table 1). 

**Figure 1:**
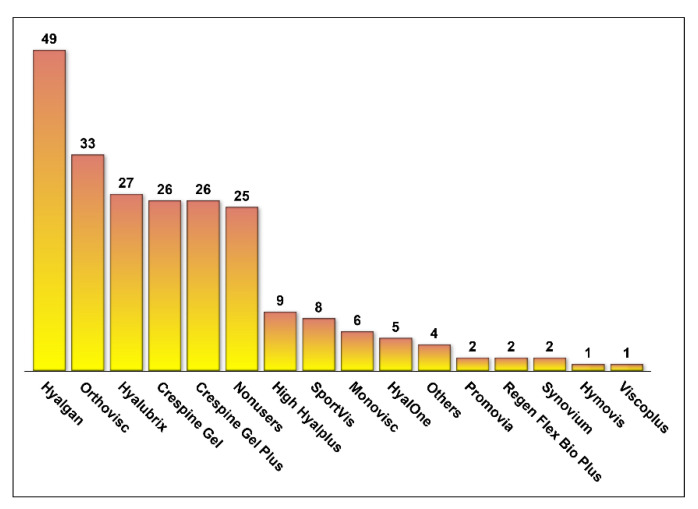
The frequency of utilizing hyaluronic acid products for osteoarthritis.

**Figure 2:**
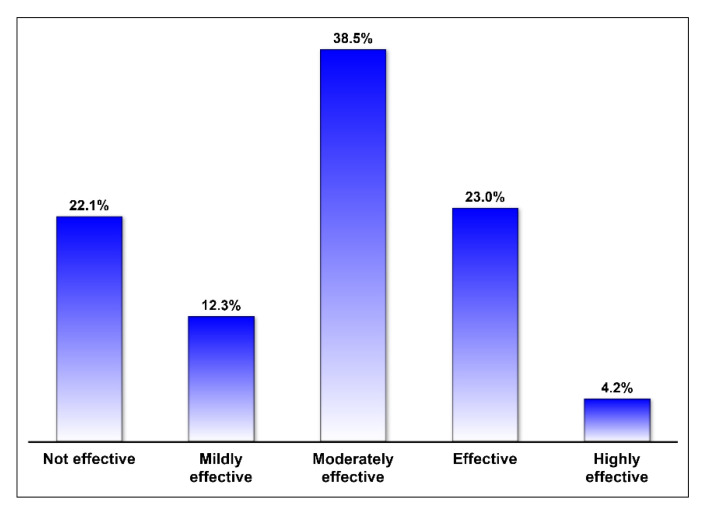
The rating of hyaluronic acid in the management of osteoarthritis.

**Figure 3:**
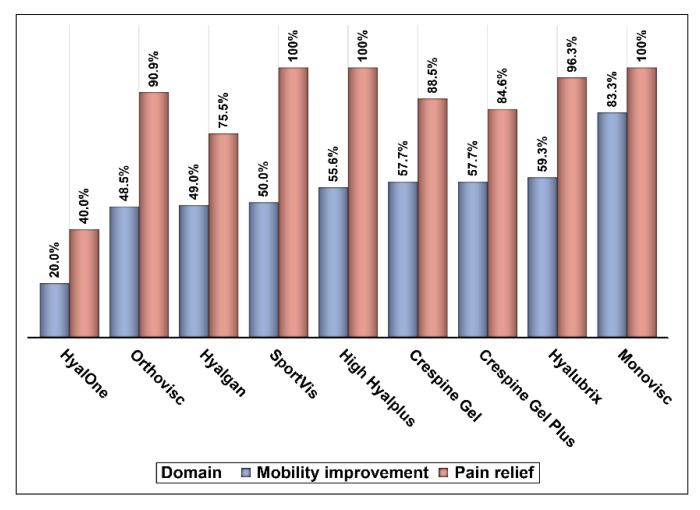
The effectiveness ratings of hyaluronic acid products.

**Figure 4:**
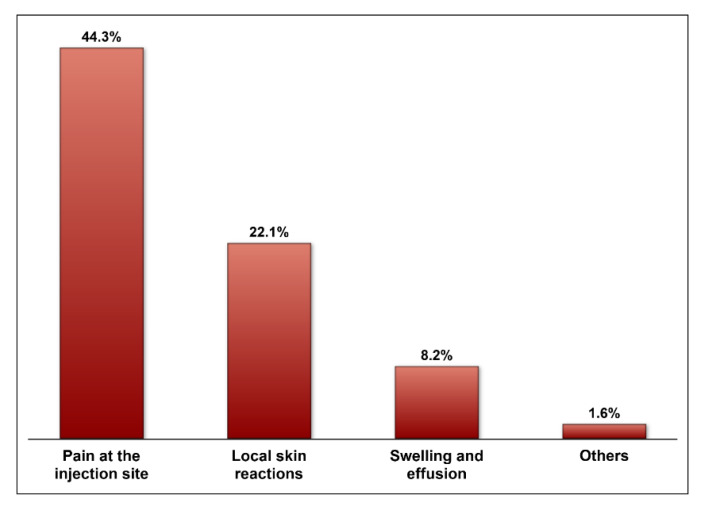
The commonly reported side effects of hyaluronic acid injections.

The majority of respondents reported using intra-articular HA products in the management of knee OA among their patients (79.51%). Hyalgan^®^, Orthovisc^®^, Hyalubrix^®^, Crespine Gel^®^, and Crespine Gel Plus^®^ were the most commonly used HA products, as shown in Figure 1. Only 27.2% of the respondents reported that intra-articular HA injections were highly effective or effective, 34.4% reported that they were either mildly effective or ineffective, and 38.5% reported that they were moderately effective, as shown in Figure 2. Intra-articular HA products that were rated for their efficacy in pain reduction and mobility improvement are shown in Figure 3. With the exception of HyalOne^®^, all rated HA products (Orthovisc^®^, Hyalgan^®^, Sportvis^®^, High Hyalplus^®^, Crespine Gel^®^, Crespine Gel Plus^®^, Hyalubrix^®^, and Monovisc^®^) were reported to relieve knee OA pain by at least 75% of the raters. However, these products did not receive similar positive ratings regarding their efficacy in improving physical function or mobility as they had in pain relief, as shown in Figure 3. Minor transitional side effects, such as pain at the site of injection, local skin reactions, swelling and effusion, and other side effects, such as inflammation at the site of injection and mild fever, were reported by the respondents as shown in Figure 4. 

## 4. Discussion

The use of intra-articular HA injections in the management of knee OA is prevalent in orthopedic practice [16]. This was confirmed among the surveyed sample of orthopedic surgeons in this study in which more than two-thirds of them reported using HA to relieve pain and improve physical function among their knee OA patients. However, the reported HA efficacy in managing pain and improving physical function was variable since less than one-third of the respondents reported that the intra-articular HA injections were effective or highly effective. This is in line with the controversial and conflicting results in the literature [13,14,21,22]. Although multiple studies have shown that HA results in significant improvement in physical function and pain management, these results were not confirmed in other systematic reviews and meta-analyses of randomized controlled trials [13,23]. Nonetheless, about two-thirds of respondents (65.7%) reported that the intra-articular HA injections were moderately effective to highly effective in managing knee OA, and only 22.1% reported that they were not effective. This suggests that the intra-articular HA injections can be useful and should be considered in the management of OA, which is consistent with some clinical guidelines for the management of OA. Additionally, the published patient-reported outcomes on the use of intra-articular HA injections for the management of knee OA are positive [24,25,26].

Although no substantial evidence exists so far on the superiority of certain brands of intra-articular HA injections over others, some studies have compared the clinical outcomes and costs associated with certain brands and formulations [21,27,28,29]. No significant differences in the risk of having total knee replacement were found among knee OA patients who received different intra-articular HA brands, such as Hyalgan^®^, Euflexxa^®^, Orthovisc^®^, and Supartz^®^, and were retrospectively followed over 3 years using commercial claims data in the United States. Interestingly, however, patients who received Synvisc^®^ which costs the most per injection (152.33 USD) in comparison to other brands (Hyalgan^®^, Euflexxa^®^, Orthovisc^®^, and Supartz^®^) and has the highest molecular weight (>6 MDa) had higher odds of having total knee replacement within 3 years of follow-up in comparison to Hyalgan^®^ and Supartz^®^. Moreover, Hyalgan^®^ and Orthovisc^®^ were the most commonly utilized intra-articular HA brands [21]. This is consistent with the findings of this study that found Hyalgan^®^ and Orthovisc^®^ to be the most commonly utilized intra-articular HA brands. Although the Orthovisc^®^ cost is higher than Hyalgan^®^ (66.55 USD vs. 38.57 USD per injection) in Saudi Arabia, the respondents did not report better improvement in mobility with Orthovisc^®^ in comparison with Hyalgan^®^. However, about 91% of the respondents who prescribed Orthovisc^®^ reported better pain relief in comparison with Hyalgan^®^ (e.g., 75.5%). This is similar to the findings of the Dasa et al. study, which did not report any significant difference between the compared HA brands despite the differences in their costs and source [21]. Furthermore, high molecular weight HAs, such as Monovisc^®^, were rated higher than Hyalgan^®^ in terms of their positive impact on mobility and pain relief. However, Sportvis^®^ was rated better than Orthovisc^®^ in terms of its effectiveness in improving mobility and relieving pain despite the fact that Sportvis^®^ has a lower molecular weight than Orthovisc^®^. Therefore, the molecular weight of HA does not seem to influence the rating of different HA brands, which is consistent with the preponderance of evidence that shows no effect of the molecular weight on the effectiveness of HA injections [24,26,28]. In addition, there are other HA administration techniques whose therapeutic values have not been explored yet among patients with knee OA. For example, the ultra-sound-guided injection of HA was found to be more effective and safer than the anatomically guided injection [30]. However, this comes with a significant extra cost and inconsistent outcomes, as reported by the latest Cochrane review [31].

Although this is the first study to the best of our knowledge that explored the value of intra-articular HA injections in the management of knee OA from the perspective of orthopedic surgeons, it has multiple limitations that must be acknowledged. First, this is a descriptive cross-sectional study that aimed to explore the views of orthopedic surgeons regarding HA injections in the management of knee OA. Thus, no causal relationship between the use of HA and pain relief or mobility improvement can be assessed. Moreover, only the views of the prescribers were considered and no patient-reported outcomes were examined. Second, convenience sampling was used which resulted in a limited generalizability of the findings. Third, only 61% of invitees participated in this survey; however, a response rate above 60% is considered good by most standards [32].

## 5. Conclusions

The use of HA in the management of knee OA was deemed moderately to highly effective in improving physical function and relieving pain based on the views of most respondents in this study. Although some respondents valued certain HA brands more favorably than others, these views were mainly subjective since differences in the molecular weight or cross-linking did not influence their views. Future studies should examine the cost-effectiveness of different HA brands due to the lack of solid evidence that demonstrates the superiority of certain brands with specific characteristics (e.g., high molecular weight or cross-linked) over other cheaper HA brands.

## Figures and Tables

**Table 1:** Baseline characteristics of the participants (*n*= 122).

Characteristic	*n* (%)
**Country**	
Saudi Arabia	73 (59.84)
Jordan	49 (40.16)
**Age**	
30–34 years	16 (13.11)
35–44 years	51 (41.80)
45–55 years	32 (26.23)
>55 years	23 (18.85)
**Years of experience**	
1–5	13 (10.66)
5–10	25 (20.49
>10	84 (68.85)
**Gender**	
Male	120 (98.36)
Female	2 (1.64)
**Postgraduate training**	
North America (e.g., United States, Canada)	17 (13.93)
Europe (e.g., France, Germany, United Kingdom)	22 (18.03)
Middle East (e.g., Saudi Arabia, Jordan, Egypt, Syria, Bahrain)	83 (68.03)
**Type of practice**	
Public	80 (65.57)
Private	42 (34.43)

## Data Availability

The data are available upon reasonable request from the corresponding author.

## Abbreviations

HA, hyaluronic acid; OA, osteoarthritis; WOMAC, Western Ontario and McMaster Universities Arthritis Index questionnaires; ESCEO, European Society for Clinical and Economic Aspects of Osteoporosis and Osteoarthritis; NSAIDs, Non-Steroidal Anti-Inflammatory Drugs.

## Appendix A

This questionnaire should be filled by practicing orthopedic surgeons only. If you are NOT an orthopedic specialist, please do not fill it out. The questions below are general and no personal identifiers (e.g., name, national ID, patient information, and hospital/clinic location or name) will be collected.

1. **Your age:**
  ☐ 30–34 years
  ☐ 35–44 years
  ☐ 45–55 years
  ☐ >55 years
2. **Gender:**
  ☐ Male
  ☐ Female
3. **Which country are you practicing in?**
  ☐ Saudi Arabia
  ☐ Jordan
4. **Where did you receive your postgraduate training?**
  ☐ Middle East (e.g., Saudi Arabia, Jordan, Egypt, Syria, Bahrain)
  ☐ North America (e.g., United States, Canada)
  ☐ Western Europe (e.g., Germany, France, United Kingdom)
  ☐ Others: ________________________
5. **How many years of experience do you have in orthopedic practice?**
  - 1–5 years
  - 5–10 years
  - >10 years
6. **Where do you primarily practice?**
  - Public/government healthcare institutions
  - Private clinics or hospitals
7. **Do you use hyaluronic acid injections in the management of patients with osteoarthritis?**
  ☐ Yes    ☐ No
8. **Please rate the efficacy of hyaluronic acid injections in the management of osteoarthritis:**
  ☐ Not effective   ☐ Mildly effective   ☐ Moderately effective
  ☐ Effective   ☐ Highly effective
9. **The following are some examples of hyaluronic acid injections that are used in the management of osteoarthritis. Please let us know whether you are using any of them by checking the box and let us know whether the hyaluronic acid products that you are using improve mobility and/or relieve pain by checking the appropriate boxes. Please note that you can check multiple boxes.**
  ☐ **HyalOne^®^**
    - Does it improve patient mobility?
      ☐ Yes    ☐ No
    - Does it relieve pain?
      ☐ Yes    ☐ No
  ☐ **Orthovisc^®^**
    - Does it improve patient mobility?
      ☐ Yes    ☐ No
    - Does it relieve pain?
      ☐ Yes    ☐ No
  ☐ **Hyalgan^®^**
    - Does it improve patient mobility?
      ☐ Yes    ☐ No
    - Does it relieve pain?
      ☐ Yes    ☐ No
  ☐ **SportVis^®^**
    - Does it improve patient mobility?
      ☐ Yes    ☐ No
    - Does it relieve pain?
      ☐ Yes    ☐ No
  ☐ **High Hyalplus^®^**
    - Does it improve patient mobility?
      ☐ Yes    ☐ No
    - Does it relieve pain?
      ☐ Yes    ☐ No
  ☐ **Crespine Gel^®^**
    - Does it improve patient mobility?
      ☐ Yes    ☐ No
    - Does it relieve pain?
      ☐ Yes    ☐ No
  ☐ **Crespine Gel Plus^®^**
    - Does it improve patient mobility?
      ☐ Yes    ☐ No
    - Does it relieve pain?
      ☐ Yes    ☐ No
  ☐ **Hyalubrix^®^**
    - Does it improve patient mobility?
      ☐ Yes    ☐ No
    - Does it relieve pain?
      ☐ Yes    ☐ No
  ☐ **Monovisc^®^**
    - Does it improve patient mobility?
      ☐ Yes    ☐ No
    - Does it relieve pain?
      ☐ Yes    ☐ No
  ☐ **Hymovis^®^**
    - Does it improve patient mobility?
      ☐ Yes    ☐ No
    - Does it relieve pain?
      ☐ Yes    ☐ No
  ☐ **Viscoplus^®^**
    - Does it improve patient mobility?
      ☐ Yes    ☐ No
    - Does it relieve pain?
      ☐ Yes    ☐ No
  ☐ **Synovium^®^**
    - Does it improve patient mobility?
      ☐ Yes    ☐ No
    - Does it relieve pain?
      ☐ Yes    ☐ No
  ☐ **Regen Flex Bio Plus^®^**
    - Does it improve patient mobility?
      ☐ Yes    ☐ No
    - Does it relieve pain?
      ☐ Yes    ☐ No
  ☐ **Promovia^®^**
    - Does it improve patient mobility?
      ☐ Yes    ☐ No
    - Does it relieve pain?
      ☐ Yes    ☐ No
  ☐ **Others: _____________________**
    - Does it improve patient mobility?
      ☐ Yes    ☐ No
    - Does it relieve pain?
      ☐ Yes    ☐ No
10. **Which of the following side effects have you noticed in patients treated with hyaluronic acid injections for osteoarthritis?**
  ☐ Pain at the injection site
  ☐ Swelling and effusion
  ☐ Local skin reactions (rash, ecchymosis)
  ☐ Others: _______________________________________________
